# ROS accumulation-induced tapetal PCD timing changes leads to microspore abortion in cotton CMS lines

**DOI:** 10.1186/s12870-023-04317-5

**Published:** 2023-06-12

**Authors:** Jinlong Zhang, Li Zhang, Dong Liang, Yujie Yang, Biao Geng, Panpan Jing, Yunfang Qu, Jinling Huang

**Affiliations:** College of Agriculture, Shanxi Agricultural University, Taigu, 030801 Shanxi China

**Keywords:** Cytoplasmic male sterility, Tapetal PCD, ROS, ROS scavenging enzyme

## Abstract

**Background:**

Cytoplasmic male sterility (CMS) is the basis of heterosis exploitation. CMS has been used to hybrid production in cotton, but its molecular mechanism remains unclear. CMS is associated with advanced or delayed tapetal programmed cell death (PCD), and reactive oxygen species (ROS) may mediate this process. In this study, we obtained Jin A and Yamian A, two CMS lines with different cytoplasmic sources.

**Results:**

Compared with maintainer Jin B, Jin A anthers showed advanced tapetal PCD with DNA fragmentation, producing excessive ROS which accumulated around the cell membrane, intercellular space and mitochondrial membrane. The activities of peroxidase (POD) and catalase (CAT) enzymes which can scavenge ROS were significantly decreased. However, Yamian A tapetal PCD was delayed with lower ROS content, and the activities of superoxide dismutase (SOD) and POD were higher than its maintainer. These differences in ROS scavenging enzyme activities may be caused by isoenzyme gene expressions. In addition, we found the excess ROS generated in Jin A mitochondria and ROS overflow from complex III might be the source in parallel with the reduction of ATP content.

**Conclusion:**

ROS accumulation or abrogation were mainly caused by the joint action of ROS generation and scavenging enzyme activities transformation, which led to the abnormal progression of tapetal PCD, affected the development of microspores, and eventually contributed to male sterility. In Jin A, tapetal PCD in advance might be caused by mitochondrial ROS overproduction, accompanied by energy deficiency. The above studies will provide new insights into the cotton CMS and guide the follow-up research ideas.

**Supplementary Information:**

The online version contains supplementary material available at 10.1186/s12870-023-04317-5.

## Background

Cotton is not only an important crop providing fiber, oil and grain, but also a strategic resource for the textile chemical industry [1]. In recent years, it is crucial to increase cotton production in view of the reduction of cultivation and increase of consumption. Like other crops such as wheat, rice, soybean and barley, cotton has obvious heterosis, and the yield and fiber quality can be improved effectively by utilizing this heterosis [2–8]. CMS is the core for the production of hybrid seeds, and it is also one of the hot spots in the field of genetics [4]. Currently, some cotton sterile lines have been developed and researched such as DBA/ZBA (CMS-D2), Zhong41A (CMS-D8) and H276A [9–15]. With the successful construction of cotton sterile, maintainer and restorer lines, the “three-line” cross breeding system have been successfully used in cotton breeding [16]. However, with just a single source and a limited number of restorers, the application of this system is greatly limited now [12, 17, 18]. Jin A and Yamian A, cultivated and studied in our laboratory, are of great significance to broaden the CMS resources of cotton [19, 20]. Jin A derived from triple hybrids [(*G. hirsutum*) × (*G. thurberi*)] × [(*G. arboreum*) × (*G. hirsutum*)] was a CMS line with *G.hirsutum* cytoplasm. It was developed through consecutive backcross procedures with the recurrent parent maintainer Jin B containing a normal fertile upland cotton (AD1) cytoplasm [19, 21]. Yamian A derived from triple hybrids [(*G. arboreum*) × (*G. bickii*)] × (*G. hirsutum)* was a CMS line with *G. arboreum* cytoplasm and the genetic background of wild *G. bickii* in Australia. Yamian B is the homotype maintainer of Yamian A [20, 22]. Our previous study indicated that the sterility rates of Jin A and Yamian A were both 100%, and their male sterility mechanisms have not been elucidated [21, 22].

As a complex process, anther development includes the proliferation and differentiation of pollen bursa multilayers, specific cells apoptosis, microsporocyte meiosis, microspore proliferation and development. This series of changes are strictly regulated in time and space [23]. As the innermost sporophytic layer, tapetum plays a major role in microspore formation [24]. It first donates proteins, lipids for microspore growth, and then supplies enzymes for micropore release [25]. After that, it needs to undergo a PCD process, making room for pollen development and depositing components [26–28]. The typical features of PCD include cytoplasmic contraction, ER expansion, nuclear membrane rupture and DNA fragmentation [28]. Abnormal tapetal PCD will lead to male sterility. Overexpression of *bHLH142* (*OE142*) triggered the early onset of PCD, leading to male sterility in rice [29]. In SaNa-1A CMS line of *Brassica napus* L., tapetal cells abnormal development and delayed degradation inhibited microspore growth [30].

In recent years, more and more genes, regulators and metabolic processes related to male sterility have been revealed through the application of new research technologies e.g., high-throughput sequencing, metabolomics, transcriptomics, proteomics, methylome and miRNAomics. These genes and processes include *miR2119b*, *FAX1* (*Fatty Acid Export 1*), *germin-like protein* (*GhGLP4*), *OsSPL*, chalcone-flavononeisomerase, pectinesterase, UDP-glucose pyrophosphorylase, starch and sugar metabolism, ATP producing, ROS scavenging and flavonoid biosynthesis [21, 31–39]. Many studies suggested that pollen abortion was associated with ROS abnormity and energy deficiency [40–45], and ROS burst was revealed in some CMS types of cotton [12, 46, 47].

ROS plays a key role in tapetal PCD, of which an extraordinary accumulation or removal will lead to abnormal PCD [25–27]. There are three main manifestations. Firstly, excessive ROS accumulation in sterile lines may affect the normal development of microspores and accompany with early degradation of tapetum, such as Kenaf CMS line 722HA and wheat YS3038-A [42, 48]. Secondly, ROS remains at a continuously low level throughout microspore development, so that tapetal degradation is abolished or delayed, as shown in rice *Defective Tapetum Death1* (*DTC1*) anthers, *Arabidopsis thaliana Respiratory burst oxidase homology* (*RBOH*) mutant and Wheat *male-sterile2* line [49–51]. Thirdly, the plant anthers show excessive accumulation of ROS but with delayed PCD in the tapetum. For example, male sterile anthers of peach due to the decrease of antioxidant content led to ROS burst, resulting in abnormal microspore and tapetum development (delayed) [52]. Excessive ROS accumulation and lack of an antioxidant enzyme system in *Brassica Napus* CMS line SaNa-1A resulted in the accumulation of malondialdehyde (MDA) in anthers, but tapetum showed delayed degradation [30].

In fact, the generation and removal of ROS in plants are in a dynamic balance. ROS can originate from various subcellular sources, including mitochondria, chloroplasts, and plasma membrane-associated NADPH oxidases [53]. As the center of plant energy metabolism, ROS can spill out by electron leakage through mitochondrial electron transport chain (mETC) complex I and III to form superoxide anion (${\mathrm{O}}_{2}^{-\bullet }$) [54]. The removal of ROS is completed by oxidoreductase, among which the most classic ROS scavenging systems are SOD, POD and CAT. They can gradually divide ROS into water and oxygen [55].

For normal pollen development, a burst of ROS is required to initiate normal PCD in tapetum which comes from NADPH oxidase [50, 56, 57]. However, in CMS anther, the source of excess ROS has not been reported. Contrary to that is there are many studies on the active enzymes in the ROS scavenging system. The absence of enzymatic and non-enzymatic ROS scavenging systems in soybean sterile line could not effectively remove ROS from anthers, and the excessive accumulation triggered PCD and eventually led to pollen abortion [40]. In wheat CMS, ROS scavenging enzyme activity increased rapidly but non-enzymatic antioxidant activity down-regulated. The balance of antioxidant system was broken, thus affecting microspore development and eventually leading to male sterility [58].

While ROS dynamics during anther development suggests ROS involves in tapetal PCD and different types of CMS are associated with ROS metabolism, it remains unclear whether ROS production unusually accumulates, whether tapetal PCD abnormality matches the failure of ROS temporal changes, and whether the ROS clearance system changes during anther development in cotton CMS lines. Here we characterized the tapetal PCD features, monitored the temporal changes of ROS and detected ROS scavenging enzyme activities during cotton pollen abortion using Jin A-CMS and Yamian A-CMS lines. In addition, we investigated the source of excessive ROS in Jin A. Our work will provide insights into the abnormal PCD in tapetum caused by metabolic imbalance in ROS.

## Results

### Tapetum morphological features in Jin A and Yamian A CMS lines

**Fig. 1:**
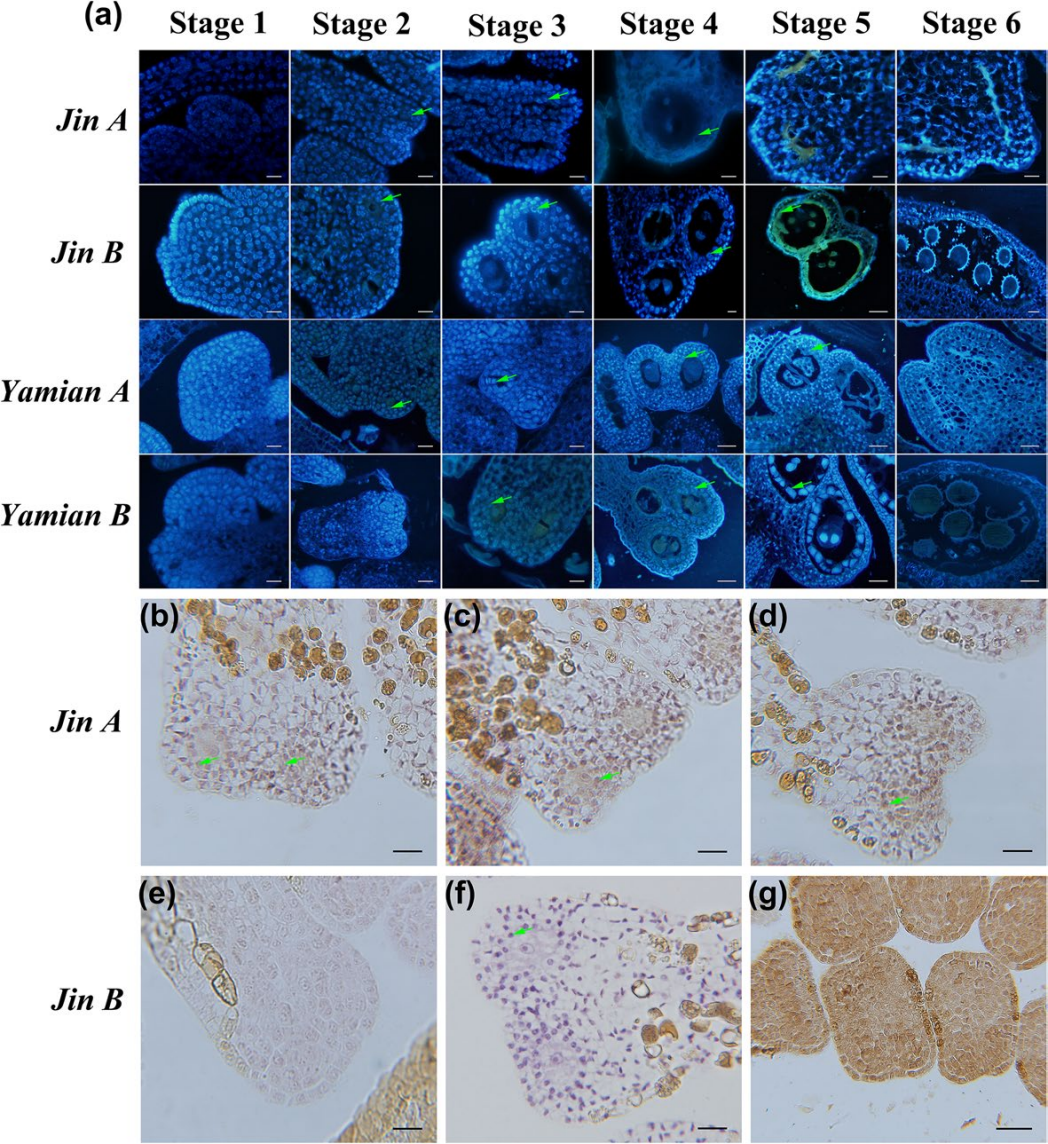
Characterization of stamen layer cells PCD. **a** Stamen paraffin sections stained with DAPI. Scale bar in Jin A at all stages, Jin B from Stage 1 to 4, Yamian A and Yamian B from Stage 1 to 3 = 20 μm, Scale bar in Jin B at Stage 5 and 6, Yamian A and Yamian B  from Stage 4 to 6 = 50 μm. The arrows showed tapetal cells. No obvious differences in 4 materials anthers at Stage 1. Compared with the fertile material anthers at Stage 2, Jin A sporogenous and some tapetal cells manifested degradation phenomena, and Yamian A showed blurred nuclei. Then the cells in Jin A anther locules were dispersed, microsporocytes fragmented, even some disappeared, and the tapetal nucleus blurred and severely degradated (Stage 3–5). Residual fragments were contained in Yamian A sporangium and non-degradable tapetum development pattern was similar to outer cells (Stage 3–5). **b **to** g** TUNEL testing in Jin A at Stage 2 (**b**) and 3 (**c** and **d**) and its maintainer Jin B at Stage 2 (**f**) and 3 (**f**) and positive control (**g**) with scale bar = 20 μm. No apoptosis in fertile anther locules (**e** and **f**), but Jin A tapetum showed DNA fragmentation at Stage 3 (**c** and **d**)

To delve into their abortive characteristics, we performed trypan blue to stain the anther tissue and 4',6-diamidino-2-phenylindole (DAPI) to dye paraffin sections. Trypan blue staining suggested that some anther cells died from Stage 4 to 6 in Jin A and Yamian A (Additional file 1: Figure 1), and DAPI showed PCD trace (Fig. 1a).

The anthers of Jin A and Jin B appeared normal at Stage 1 (Fig. 1a). They all differentiated into sporangia in the corner epidermis of the stamen primordium, and the nucleus of sporogonium was significantly larger than peripheral tissue cells. However, at Stage 2 when three layers of cells formed in the sporangia with larger nuclei in Jin B, Jin A sporogenous and some tapetal cells manifested degradation phenomena such as different nuclear size and fuzziness. Thereafter, normal anther locules divided 4 layers, where microsporocytes were surrounded by the epidermis, endothecium, middle layer and tapetum, but in Jin A, the anther locules were dispersed, microsporocyte fragmented, even some disappeared, and tapetal nucleus blurred and severely degraded (Fig. 1a Stage 3). During Stage 4, microspore mother cells began meiosis and tapetum contracted inward with binuclear in Jin B anther, but sterile line pollen loculus were empty or had only a small number of cell residues and tapetal cells had degenerated and disappeared. Thereafter, Jin B microspore mother cells underwent normal meiosis to form tetrad; tapetum secreted enzymes, separated the tetrad, and itself gradually blurred degradation; eventually the microspores matured and formed pollen grains with spiny processes, and the tapetum was completely degraded, only represented residual partial traces (Fig. 1a Stage 5 and 6). Contrastingly, the sporangia in Jin A contracted after the meiosis, outer cells conducted mitosis, filled the entire sporangia (Fig. 1a Stage 5), and finally formed anthers without pollen grains (Fig. 1a Stage 6). To further characterize the tapetal PCD from Stage 2 to Stage3 in Jin A, DNA fragmentation was detected using TUNEL assay. There was no brown TUNEL signal in fertile anther locules at Stage 2 and 3 (Fig. 1e and f). However, a weak TUNEL signal was detected in Jin A microspore mother cells and tapetal cells at Stage 2 (Fig. 1b), thereby indicating that PCD were present in this stage. At Stage 3 Jin A cells produced stronger brown TUNEL-positive signals which was explained by the obvious accumulation of DNA cleavage (Fig. 1c, d, and g was positive control).

**Fig. 2:**
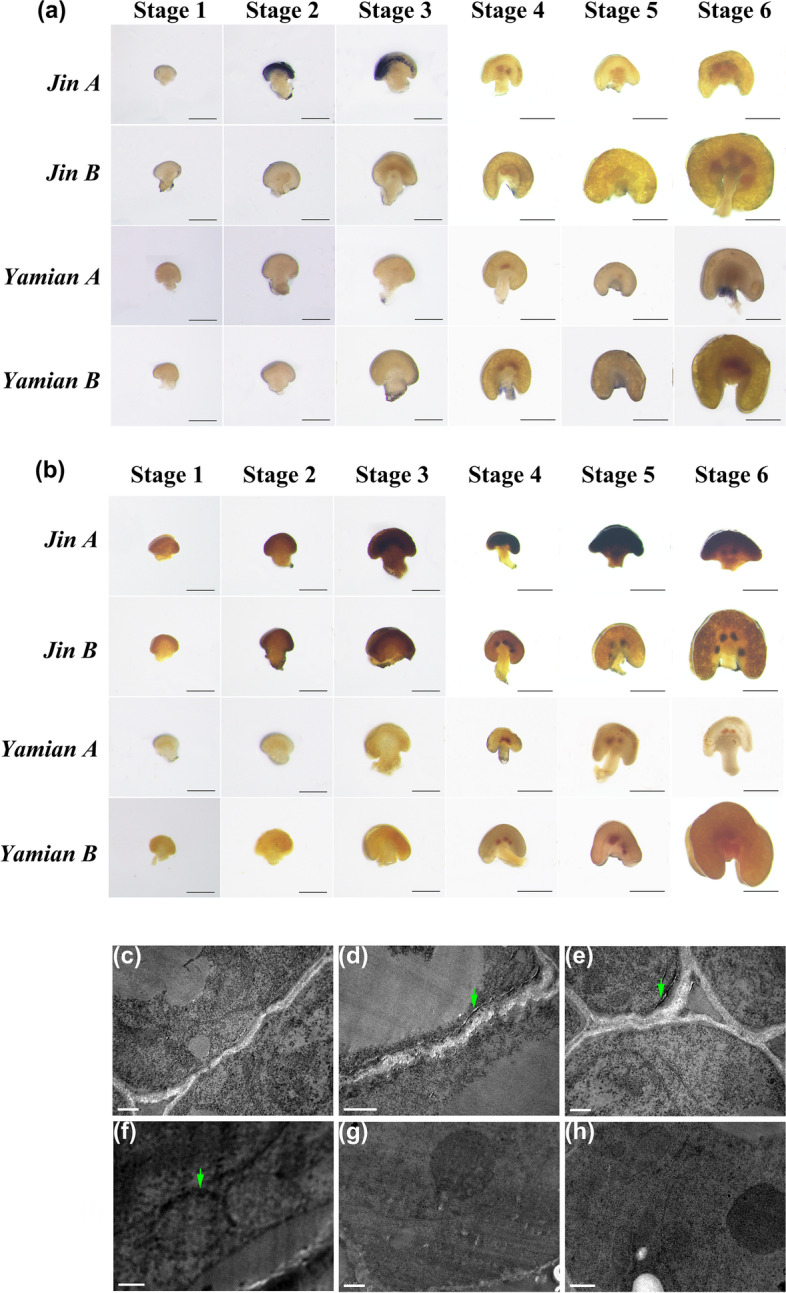
Comparison of ${\mathrm{O}}_{2}^{-\bullet }$ and H_2_O_2_ levels. **a** Stamen stained with NBT. Scale bar at Stage 1 and 2 = 200 μm, Stage 4, 5 and 6 = 500 μm. Jin A ${\mathrm{O}}_{2}^{-\bullet }$ gathered at Stage 2 and 3; Yamian A showed no ${\mathrm{O}}_{2}^{-\bullet }$ enrichment. **b** Stamen stained with DAB. Scale bar at Stage 1 and 2 = 200 μm, Scale bar at Stage 4, 5 and 6 = 500 μm. Jin A H_2_O_2_ gathered from Stage 3 to 6; Yamian A showed no H_2_O_2_ enrichment. **c **to** h** Stamen H_2_O_2_ subcellular localization between Jin A at Stage 2 (**c**), Stage 3 (**d**, **e** and **f**) and Jin B at Stage 2 (**g**), Stage 3 (**h**). Scale bar in **c**, **e**, **g** and **h** = 0.5 μm, **d** = 1 μm, and **f** = 0.25 μm. The arrows showed H_2_O_2_ deposition. No H_2_O_2_ deposition in Jin B at Stage 2 and 3, but obvious sedimentary H_2_O_2_ in Jin A at Stage 3

The anther development characteristic of Yamian B was the same to Jin B. The microspore mother cells of Yamian A showed blurred nuclei at Stage 2, and thereafter only residual fragments were contained in the sporangium (Fig. 1a Stage 3–5), which was eventually filled with outer cells (Fig. 2b Stage 6). The difference of tapetum mainly occurred at Stage 4, which were characterized by the absence of nuclear enlargement, binuclear and condensation, and the development pattern was similar to outer cells.

These results indicated that abnormal development of microspores in Jin A occurred simultaneously with tapetum degradation, and the early PCD of tapetum was related to pollen abortion. Yamian A also showed abnormal microspore development at Stage 2, but tapetum no degradation or delayed PCD.

### Accumulation of ROS in Jin A and Yamian A

To explore the relationship between abnormal anther tapetal PCD and ROS metabolism, we determined ${\mathrm{O}}_{2}^{-\bullet }$, H_2_O_2_ as well as MDA content. Nnitrotetrazoliumblue chloride (NBT) dyeing ${\mathrm{O}}_{2}^{-\bullet }$ showed that compared with Jin B, the anther tissue was obviously stained blue at Stage 2 and 3 (Fig. 2a) in Jin A, indicating palpable ${\mathrm{O}}_{2}^{-\bullet }$ accumulation at that time. We also determined the ${\mathrm{O}}_{2}^{-\bullet }$ content of flower buds (Additional file 2: Figure 2a). Great increases occurred at Stage 2 and 3 in Jin A which was consistent with the results of NBT staining, illustrating ${\mathrm{O}}_{2}^{-\bullet }$ accumulation was abnormal in the critical period of pollen abortion. 3,3’-Diaminobenzidine (DAB) staining H_2_O_2_ showed that compared with Jin B, the anthers at Stage 3 and later stages showed obvious brown color, indicating obvious accumulation of H_2_O_2_ at that time (Fig. 2b). And the content of H_2_O_2_ (Additional file 2: Figure 2b) were consistent with the results of DAB staining, illustrating the abnormal accumulation of H_2_O_2_ in Jin A during the critical period of pollen development. In addition, MDA content was similar to H_2_O_2_, and higher at Stage 3 (Additional file 2: Figure 2c).

The H_2_O_2_ accumulated in cells can be stained by cerium chloride to form electron-dense precipitation, which can be observed by an electron microscope. Jin B showed obvious nucleolus, clear nuclear membrane, nuclear pores in some positions and complete mitochondrial and no palpable H_2_O_2_ electron-dense black precipitate was found in mitochondrial and cell membrane at Stage 2 (Fig. 2g). Jin A tapetum showed signs of degeneration, nucleolus disintegrate and nuclear membrane blurred, and no electron dense black precipitate or just a bit was found in cell membrane or other parts (Fig. 2c), indicating that H_2_O_2_ accumulation was not found at this time. However, at Stage 3, compared with no obvious H_2_O_2_ deposition (Fig. 2h) found in fertile line, Jin A tapetum showed significant H_2_O_2_ electron-dense black precipitates, which appeared around the cell membrane (Fig. 2d and e), intercellular space (Fig. 2e), and mitochondrial membrane (Fig. 2f).

But Yamian A showed a contrary result. In both DAB and NBT staining, the anther tissue of Yamian A was not colored like Yamian B (Fig. 2a and b). However, the content of ${\mathrm{O}}_{2}^{-\bullet }$, H_2_O_2_ and MDA were significantly lower during the critical period of microspore abortion (Additional file 2: Figure 2a and b), implying the low level of ROS in Yamian A (Additional file 2: Figure 2c).

Based on these results, we suggested that microspore abortion had correlation with the accumulation of ROS in Jin A. The level of ROS increase might lead to cell metabolism imbalance and eventually infertility. H_2_O_2_ accumulation in tapetum might be the cause of apoptosis and early degradation. The low ROS level in Yamian A during microspore abortion might be the reason for delayed or non-degradation of tapetum.

### Enzymatic activities of ROS-scavenging

**Fig. 3:**
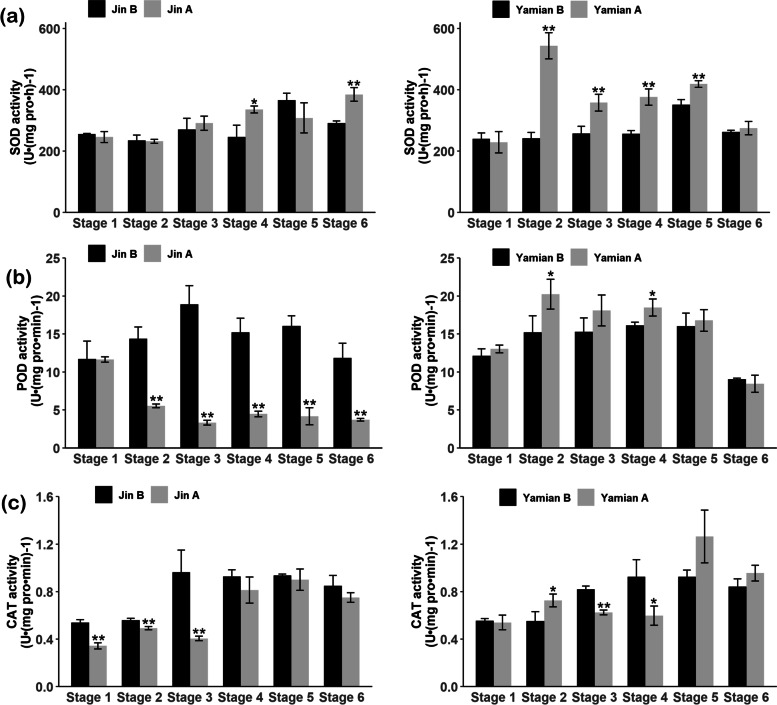
Determination of SOD (**a**), POD (**b**) and CAT (**c**) activities in Jin A (left), Yamian A (right) and their maintainers Jin B, Yamian B. Values are means ± SD of three replicates. Asterisks represent statistically significant differences between sterile line and its maintainer (* *P* < 0.05; ** *P* < 0.01, Student’s t tests). SOD, superoxide dismutase; POD, peroxidase; CAT, catalase

The concentration of ROS is determined by the composition and availability of antioxidant systems. We measured the activities of enzymes including SOD, POD and CAT involved in ROS scavenging. The results were as follows: For the flower buds of Jin A, SOD activity increased only between Stage 4 and Stage 6, when microspore abortion and tapetum degradation accomplished, and there was no significant difference in other stages (Fig. 3a). POD activity was significantly lower than Jin B except Stage 1 (Fig. 3b). CAT activity decreased at Stage1, 2 and 3, and showed no difference after microspore abortion and tapetum degradation (Fig. 3c). For Yamian A, SOD activity increased significantly from Stage 2 to 5 (Fig. 3a). POD activity at Stage 2 and 4 was significantly higher than Yamian B (Fig. 3b). CAT activity was significantly higher at Stage 2, but significantly lower at Stage 3 and 4 (Fig. 3c).

In summary, the decrease of POD and CAT activities seemed to be more closely related to the accumulation of ROS, while the increase of SOD and POD activities was responsible for the decrease of ROS content in Yamian A.

SOD, CAT and POD enzymes contain a variety of isoenzymes and they represent differences in biological activity and even functional diversity at different parts of cells [59–63]. In Jin A, there were no significant differences in the expression of *Cu-ZnSOD* and *FeSOD* from Stage 2 to 3, and the expression trend of *MnSOD* is the same as SOD activity (Additional file 3: Table 1). The *GPX6* and *CAT2* expression level was lower in Jin A which were consistent with POD and CAT activities. In Yamian A, *Cu-ZnSOD* and *APX* expressions were higher, and *CAT1* was consistent with CAT activity. Therefore, the differences of ROS elimination enzymes activities may be related to the expression changes in various isoforms.

### ROS origin

NADPH oxidase was widely considered as the main source of ROS burst/generation in anther, especially *RBOHE* [48, 55, 56]. Therefore, we tested the expression of *RBOHE* (Additional file 4: Table 2). Interestingly and amazingly, the expressions were both down-regulated in Jin A and Yamian A indicating *RBOHE* representing less effect for ROS generation.

**Fig. 4:**
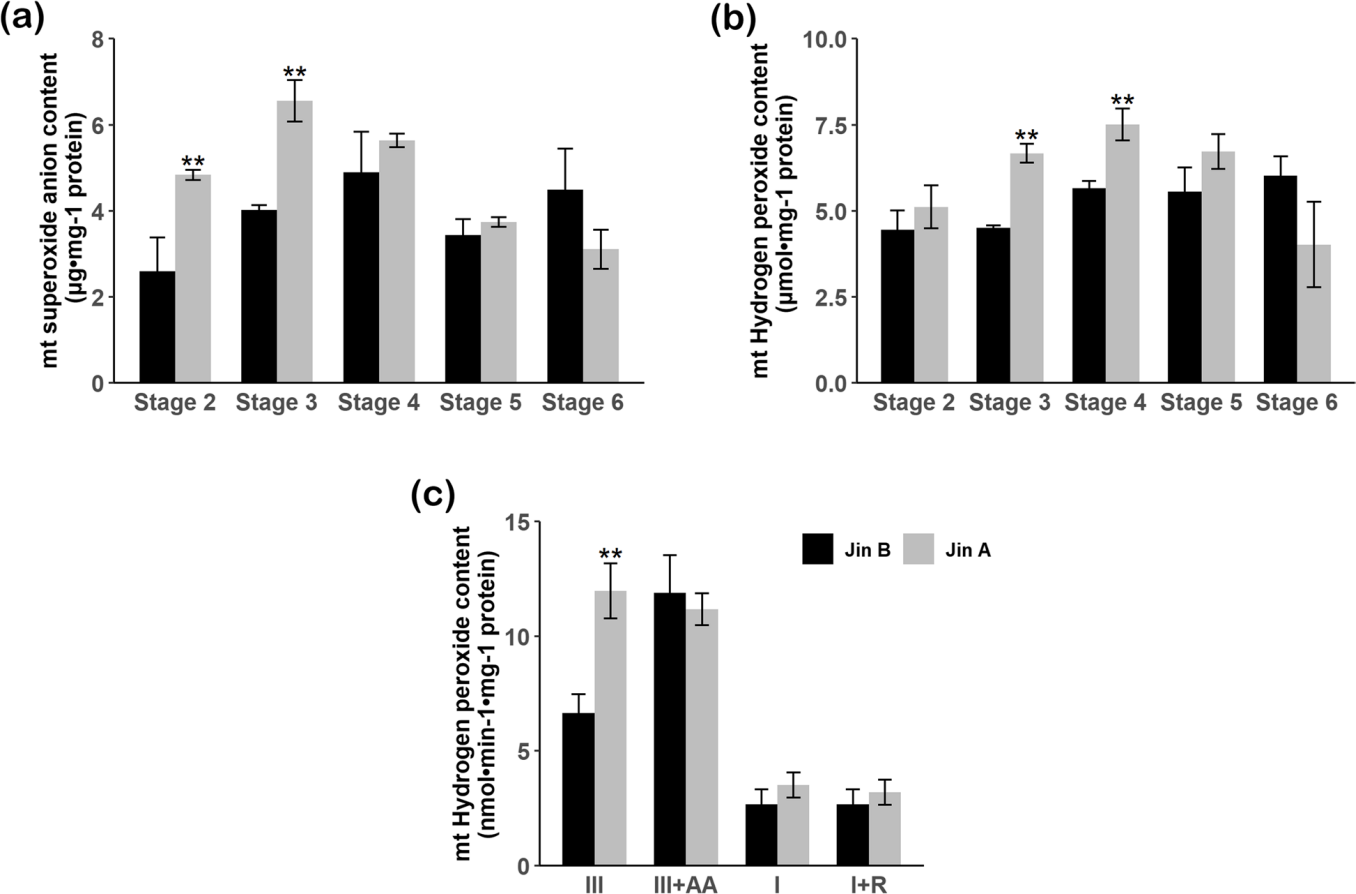
Comparison of mitochondrial ROS levels between Jin A and its maintainer Jin B. **a** Content of flower buds mitochondrial ${\mathrm{O}}_{2}^{-\bullet }$ between Jin A and Jin B. **b** Content of flower buds mitochondrial H_2_O_2_ between Jin A and Jin B. **c** Production of ${\mathrm{O}}_{2}^{-\bullet }$ by Jin A and Jin B etiolated seeding submitochondrial particles. Values are means ± SD of three replicates. Statistical differences are the same as Fig. 3. I, Complex I; III, Complex III; AA, Antimycin A; R, Rotenone

**Fig. 5:**
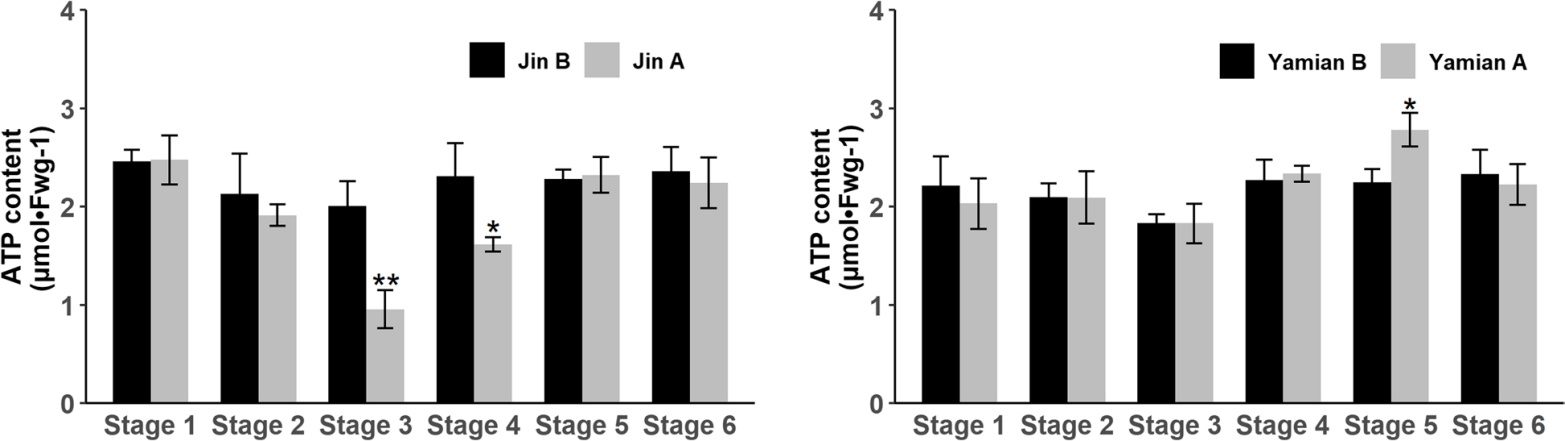
Determination of ATP content in Jin A (left), Yamian A(right) and their maintainers Jin B, Yamian B. Values are means ± SD of three replicates. Statistical differences are the same as Fig. 3

Given the results of *RBOHE* expression and H_2_O_2_ subcellular localization we identified the mitochondrial ROS. Therefore, we detected ROS content in isolated mitochondria. During the critical period of Jin A pollen abortion, it exhibited significantly higher ${\mathrm{O}}_{2}^{-\bullet }$ (Fig. 4a) and H_2_O_2_ (Fig. 4b) than Jin B. At the same time, we tested the generation rate of ${\mathrm{O}}_{2}^{-\bullet }$, and found that ${\mathrm{O}}_{2}^{-\bullet }$ from mitochondrial complex I had no differences between Jin A and Jin B regardless of whether adding rotenone or not (Fig. 4c). However, when inhibitor absence, ${\mathrm{O}}_{2}^{-\bullet }$ generation from complex III was significantly higher than Jin B (Fig. 4c), and this change was abolished when adding antimycin A. The results supported Jin A complex III might produce excess ROS in normal condition. In addition, we measured the changes of ATP content. The results showed that ATP content had no differences at each stage of anther development in maintainers, while it decreased significantly at Stage 3 and 4 in Jin A (Fig. 5).

We therefore concluded that disordered mitochondrial ROS generation and accumulation may be a significant contribution to ROS anomaly during Jin A pollen abortion. Energy metabolism may be not closely related to Yamian A abnormal anther development.

## Discussion

### Microspore abortion and tapetal abnormal PCD in CMS

Normal pollen development is a continuous process. In sporangia, microspore mother cells, dividing from sporogenous cells, proliferate and subsequently undergo meiosis to form microspores, and eventually develop into mature pollen grains through two rounds of cell division. Abnormalities at any step in this process can result in microspore development failure and pollen malformation [64]. Towards cotton male sterility germplasm, photosensitive male sterile mutant anther abortion occurred at the microspore stage; GMS 1355A abortion occurred during the release of microspores; CMS H276A and zhong41A pollen mother cells were gradually dissolved at the tetrad stage [14, 15, 65, 66]. Meanwhile, it should be noted that tapetum is close to the microspores, and its appropriate PCD is important. The abnormal activities including delayed and premature PCD in tapetum will lead to the obstruction of microspore development and pollen abortion. In previous reports, premature tapetal PCD had been described in cotton *harknessii* A, C2P5A, rice WA-CMS, wheat K87B1-706A, and delayed degradation was occurring in wheat u87B1-706A and *Brassica napus* L. SaNa-1A [12, 30, 46, 58, 67]. In this research, the abnormal microspore development of Jin A and Yamian A could be traced back to Sporogenous cells stage (Stage 2) when the microspore nuclear membrane was not complete and the nucleus turned blur and degenerative. At the same stage in Jin A, tapetal cells showed degeneration and disappearance and the fragmentation of nuclear DNA (TUNEL signal) appeared. This suggested Jin A tapetal cells underwent PCD in advance, and its early disintegration was a key feature of microspore abortion. Moreover, interestingly, Yamian A showed the opposite developmental state that compared with Yamian B, no degradation of tapetum conducted at Stage 4. This difference may be determined by different abortion mechanisms in different CMS types. Therefore, Jin A and Yamian A could enrich the germplasm diversity of male sterility and provide new materials and new ideas for the study of CMS in cotton. The study of genes that regulated the early or delayed degradation of tapetum will be a goal of our future research and will be beneficial to our understanding of the CMS molecular mechanism.

### Mitochondrial ROS and abnormal energy metabolism leading to tapetum PCD

The production of ROS is an important factor in plant PCD signaling [68]. Actually, during the process of normal anthers development, the amount of ROS usually increased at the particular stage of the tapetum abortion process, and the peak time of ROS production was associated with stage-specific expression of NADPH oxidase, especially *RBOHE* [50, 56, 57]. Recent study had identified that abscisic acid (ABA) could trigger ROS burst in rice developing anthers leading to tapetal PCD when experiencing heat stress [69]. This excess ROS production in stress-induced male sterility also related to NADPH oxidase on account of ABA stimulated the hyperpolarization-activated Ca^2+^ channels and up-regulated the activity of NADPH oxidase [70]. RT-PCR analysis showed a down-regulated expression of *RBOHE* in Jin A and this strange result suggested that excess ROS in tapetum might be not produced by NADPH oxidase.

During cell death-inducing conditions, the mETC may become inhibited, plausibly producing cytotoxic levels of ROS [71, 72]. In addition, PCD induced by externally applied ROS could be mediated by mitochondria [73–76]. These findings supported a central role for mitochondria in PCD, where ROS was produced by complex I and III [63]. The accumulation of ROS includes two factors. One hand is excessive ROS produced. The main causes of CMS were the changes of key genes or generation of chimeric ones caused by mitochondrial genome rearrangement or mutation, and these genes may inhibit mETC and F_0_F_1_-ATP synthase which caused the surplus of electron transfer [77–80]. The excess electrons are converted into ROS. *orf610a* was identified as sterility gene in cotton CMD-D2 line ZBA. This chimeric gene was specifically expressed in sterile line and resulted in excessive accumulation of ROS and reduction in ATP content when ectopic expression in yeast [78]. Another is ROS scavenge or elimination. SOD、POD and CAT enzymes system is a classical one in plants to resist oxidative stress and they form a complete antioxidant chain and work together for ROS scavenge or elimination [81]. Also, these antioxidant enzymes can suppress plant PCD when specifically targeted to mitochondria [14]. In cotton CMS-D2 line, excessive H_2_O_2_ accumulated along with POD activity significantly decreasing [12]. Lack of positive regulation of SOD and its activity declining induced ROS balance disrupted in Kenaf CMS 722HA [42]. The activity levels of SOD, CAT and POD in IAMSLs were higher than the maintainer, which was the result of the activation of antioxidant system triggered by the increased production of ${\mathrm{O}}_{2}^{-\bullet }$ and H_2_O_2_ [58]. In addition, the change in various isoforms of antioxidant enzymes was closely associated with the tissue-specificity of ROS scavenge/elimination in plant organelles. In our study, compared to maintainer, Jin A complex III overflowed excess ROS. ROS cannot be cleared in time on account of POD and CAT activities decreased which caused by related isoform *GPX6* (which can be localized in mitochondria [61]) and *CAT2* (whose main function is to clear ROS [63]) transforming. These both led to a high level of ROS in mitochondrial and anther tissues. The accumulation of ROS induced the signal of PCD in tapetum and caused DNA fragmentation in parallel with ATP content sharp declined at the same time. These experimental results pointed out mitochondrial ROS and energy metabolism disorder were related to pollen abortion.

**Fig. 6:**
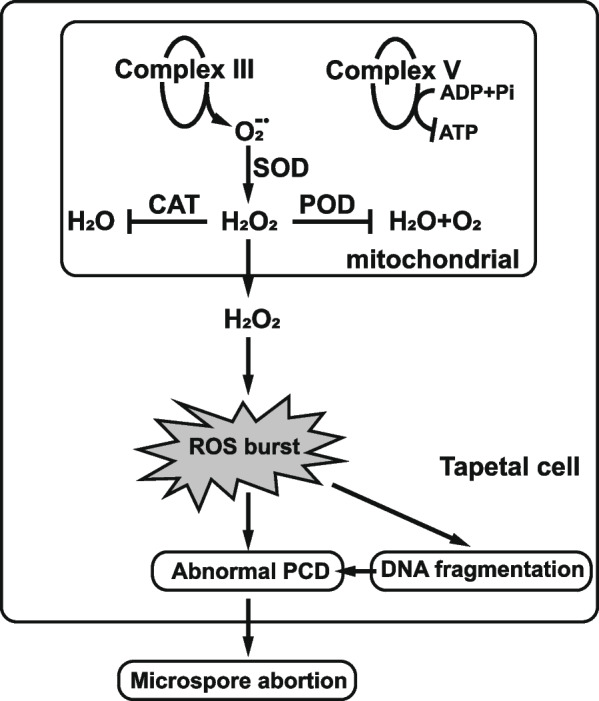
A proposed working model of pollen abortion in Jin A. In parallel with the decrease of ATP level, mitochondrial electron transport chain complex III of tapetal cells leaked out ${\mathrm{O}}_{2}^{-\bullet }$, which generates H_2_O_2_ under the action of SOD. Due to the reduced activities of POD and CAT, ROS cannot be removed in time, which leaded to ROS burst. Excessive ROS triggered DNA fragmentation and caused premature apoptosis of tapetum, and eventually resulted in microspore abortion

Based on this study, we constructed a proposed working model of impaired microspore development in Jin A (Fig. 6). In parallel with the decrease of ATP level, mitochondrial electron transport chain complex III of tapetal cells leaked out excess ${\mathrm{O}}_{2}^{-\bullet }$, which generates H_2_O_2_ under the action of SOD. Due to the reduced activities of POD and CAT, ROS cannot be removed in time and resulted in ROS burst. Excessive ROS triggered DNA fragmentation and caused premature apoptosis of tapetum, and eventually resulted in microspore abortion.

Given the phenomenon of tapetal no or delayed degradation in Yamian A, it was not surprising that ROS content was reduced in flower buds. However, there are few reports on the decrease of ROS content and delayed or non-degraded tapetum in other CMS lines, but many on regulatory factors [49, 50, 56, 57]. Yamian A down-regulated *RBOHE* expression might be related to the lower ROS, and the up-regulated *Cu-ZnSOD* and *APX* increased SOD and POD activities. In wheat, *orf279* (*ATP synthase subunit 8*) had been reported as an AL-type (AL18A) CMS gene, which resulted in delayed tapetal PCD with abnormal expression of mETC and ROS scavenging enzymes in early anther development period [79, 80]. Although the performance of Yamian A was similar to AL18A, no significant difference in ATP content suggested that Yamian A might have less relationship with mitochondrial energy metabolism, and its abortion mechanism still needs to be further studied. In addition, the difference in tapetal PCD between the two materials confirmed the core role of ROS accumulation, which will provide guidance for our future work.

## Methods

### Plant materials

All materials were planted at the Farm Station of Shanxi Agricultural University in the summer of 2021, Crop management practices followed normal recommendations. When the cotton developed to the flowering stage, the buds of different developmental stages were picked. Based on the preliminary observation of microspore development in cotton, we divided anther development into six stages [22]. After removing the bracts, the buds were divided into 6 different periods: Stage1 (Sporogonium stage), Stage 2 (Sporogenous cells stage), Stage 3 (Microsporocyte stage), Stage 4 (Meiosis stage), Stage 5 (Tetrad stage), Stage 6 (First nuclear and pollen maturation stage). The buds used in cytological experiments were treated according to the general operation, and the rest were frozen with liquid nitrogen, then stored at -80℃ refrigerator.

### Trypan blue, NBT and DAB staining

Trypan blue staining was performed as *Mou *et al*.* described and dead cells or tissues were stained blue [82]. NBT and DAB staining was performed as *Wu *et al*.* described and ${\mathrm{O}}_{2}^{-\bullet }$ and H_2_O_2_ were stained blue and brown, respectively [83].

### DAPI staining and TUNEL assays

DAPI staining was performed as routine paraffin section making process. When dyeing, added 100μL DAPI dye solution (10 μg/mL), and after 5–15 min staining at room temperature, added a drop of glycerol, and covered the cover glass before being visualized at fluorescence microscope.

TUNEL assays were performed using the TUNEL Apoptosis Detection Kit (BIOTIN marking POD method) (KeyGEN BioTECH, Jiangsu, China) according to the supplier’s instructions.

### Subcellular localization of H_2_O_2_: cerium chloride treatment

Subcellular localization of H_2_O_2_ was performed as *Luo *et al*.* described [62]. Briefly, The buds were incubated at 5 mM cerium chloride solution (dissolve in 50 mM MOPS, pH7.2) for 1 h, then fixed in 50 mM of sodium dimethylarsinate (pH7.2) with 1.25% glutaraldehyde/1.25% paraformaldehyde. After general SEM slice operation, H_2_O_2_ accumulation was observed using transmission electron microscope (Hitachi H-7500, Japan).

### Determination of ROS and MDA content

The content of ${\mathrm{O}}_{2}^{-\bullet }$ and H_2_O_2_ was determined with standard curve method in spectrophotometry.

Prepared 0.4 ml sodium nitrite standard solution (0-10 μg, dissolve in acetone), added 0.4 ml 17 mM anilinparasulfonic acid, 0.4 ml 7 mM α-naphthylamine, and then incubated at 30℃ for 30 min. The absorbance was determined at 530 nm. Samples of bud (~ 0.1 g) at different stage were ground in 1 ml PBS(65 mM, pH 7.8). The homogenates were centrifuged at 10,000 rpm for 10 min. 150 μl PBS and 50 μl hydroxylamine hydrochloride (10 mM) were added to the supernatant (0.2 ml), then incubated at 25℃ for 20 min. The absorbance value of the reaction solution was determined by the same method, and the ${\mathrm{O}}_{2}^{-\bullet }$ content was calculated according to the standard curve.

Prepared 1 ml H_2_O_2_ standard solution (20-100 μM, dissolve in acetone), and added 0.1 ml 2 M titanium sulfate. The homogenates were centrifuged at 12,000 rpm for 10 min. The supernatant then added 0.2 ml ammonia spirit (25%-28%), centrifuged at 12,000 rpm for 10 min.The precipitate added 3 ml 2 M sulfuric acid to completely dissolve. The absorbance was determined at 415 nm. Samples of bud (~ 0.1 g) were ground in acetone, and the absorbance value was determined by the same method and the H_2_O_2_ content was calculated according to the standard curve.

Blend the bud (~ 0.2 g) to a smooth paste with distilled water (5 ml), added 5 mL 0.5% thiobarbituric acid solution (dissolved in 20% trichloroacetic acid), and Boiled for 10 min. The volume was measured after filtration and the OD values at 450 nm, 532 nm and 600 nm were determined. MDA contents were calculated using the following formula:

$$\mathrm{MDA}\;\mathrm{concentration}\;\mathrm C\;(\mu\mathrm{m}\mathrm o\mathrm l/\mathrm L)=6.45\;({\mathrm A}_{532}-{\mathrm A}_{600})-{0.56\mathrm A}_{450},$$

$$\mathrm{MDA}\;\mathrm{content}\;(\mu\mathrm{m}\mathrm o\mathrm l/\mathrm g)=\mathrm C\times\mathrm N\times10^{-3}/\mathrm{Fw}$$

N, the volume of the solution after the reaction; Fw, the fresh weight of the sample.

### Determination of ROS scavenging enzyme activities

SOD, POD, and CAT enzyme activites determination were performed as *Demircan* et al. and *Bradford* et al. described [84, 85].

### Mitochondrial extraction and ROS testing

Mitochondria were extracted from various tissues or anthers using Plant mitochondrial Extraction Kit (biolab Biotechnologies Beijing) according to the manufacturer’s instructions.

${\mathrm{O}}_{2}^{-\bullet }$ and H_2_O_2_ assay were similar with described above. They were determined with standard curve method in enzyme-labeled instrument. The generation rate of ${\mathrm{O}}_{2}^{-\bullet }$ was performed as *Boveris* described [54].

#### RT-PCR

RNA extraction were performed using Plant RNA Extraction Kit (Aidlab Biotechnologies Co.,Ltd, China) according to the supplier’s instructions. Reverse transcription were performed using PrimeScript™ RT reagent Kit with gDNA Eraser (Perfect Real Time) (Takara Bio Dalian, Inc.). RT-PCR were performed using TB Green® Premix Ex Taq™ II (Tli RNaseH Plus) (Takara Bio Dalian, Inc.) at Bio-rad CFX Connect fluorescent PCR amplifier (Bio-Rad Laboratories, Inc.). Primers were shown in Additional file 5: Table 3. Primer synthesis were performed by Beijing Tsingke Biology Co., Ltd.

### Determination of ATP

TUNEL assays were performed using the ATP Detection Kit (Beijing Solarbio Science and Technology Co., Ltd) according to the supplier’s instructions.

## Supplementary Information

**Additional file 1: Figure 1.** Stamen stained with Trypan blue. Scale bar in Stage 1 and 2 =200μm, Stage 4, 5 and 6 =500μm. Jin A and Yamian A anther cells death mainly occurred at Stage 4 to 6, but in fertile lines, cell debris were present on the pollen surface at Stage 6.

**Additional file 2: Figure 2.** Determination of ${\mathrm{O}}_{2}^{-\bullet }$, H_2_O_2_ and MDA content in Jin A (left), Yamian A(right) and their maintainers Jin B, Yamian B. (a) Content of ${\mathrm{O}}_{2}^{-\bullet }$ in Jin A (left) and Yamian A (right). (b) Content of H_2_O_2_ in Jin A (left) and Yamian A (right). (c) Content of MDA in Jin A (left) and Yamian A (right). Values are means ± SD of three replicates. Asterisks represent statistically significant differences between sterile line and its maintainer (* *P* < 0.05; ** *P* < 0.01, Student’s t tests).

**Additional file 3: Table 1.** The relative expression of antioxidase genes by qRT-PCR. Values are means ± SD of three replicates. Asterisks represent statistically significant differences between sterile line and its maintainer (*P < 0.05; ** *P* < 0.01, Student’s t tests). GPX, Glutathione peroxidase; APX, Ascorbate peroxidase.

**Additional file 4: Table 2.** The relative expression of *RBOHE* by qRT-PCR. Values are means ± SD of three replicates. Asterisks represent statistically significant differences between sterile line and its maintainer(* *P* < 0.05; ** *P* < 0.01, Student’s t tests).

**Additional file 5: Table 3. **Primer sequence.

## Data Availability

All the data supporting the results in this article are included in the present and the additional files.

## Abbreviations

CMS: Cytoplasmic male sterility
PCD: Programmed cell death
ROS: Reactive oxygen species
MDA: Malondialdehyde
SOD: Superoxide dismutase
POD: Peroxidase
CAT: Catalase
mETC: Mitochondrial electron transport chain
ABA: Abscisic acid
