# Supplementary figures and images for: ROS accumulation-induced tapetal PCD timing changes leads to microspore abortion in cotton CMS lines
Source: BMC Plant Biol. 2023 Jun 12;23:311. doi: 10.1186/s12870-023-04317-5 (PMC10259065; doi:10.1186/s12870-023-04317-5)

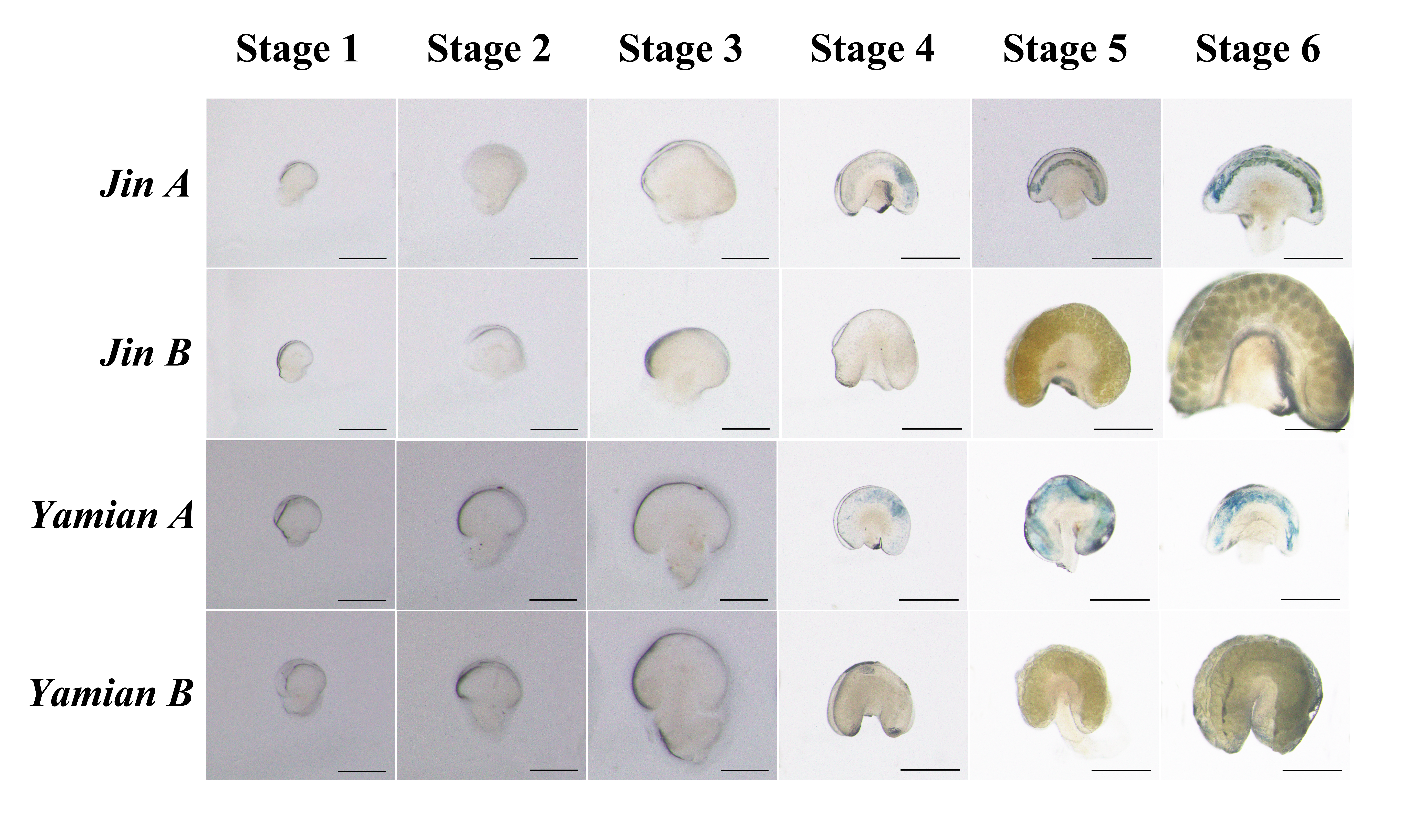

Supplement: Supplementary file 1 — Additional file 1: Figure 1. Stamen stained with Trypan blue. Scale bar in Stage 1 and 2 =200μm, Stage 4, 5 and 6 =500μm. Jin A and Yamian A anther cells death mainly occurred at Stage 4 to 6, but in fertile lines, cell debris were present on the pollen surface at Stage 6. [file 12870_2023_4317_MOESM1_ESM.tif]

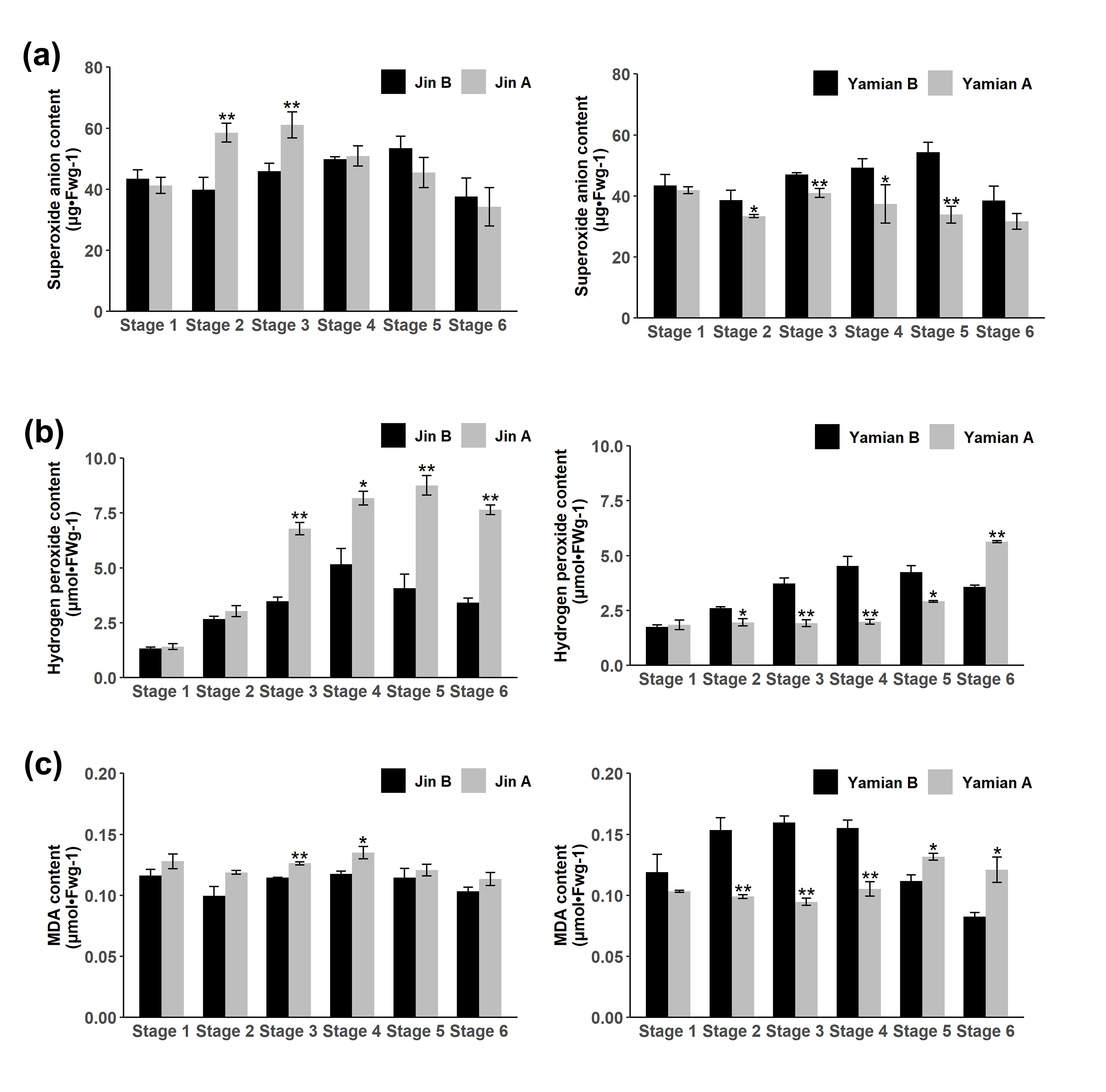

Supplement: Supplementary file 2 — Additional file 2: Figure 2. Determination of ${\mathrm{O}}_{2}^{-\bullet }$O2-∙, H2O2 and MDA content in Jin A (left), Yamian A(right) and their maintainers Jin B, Yamian B. (a) Content of ${\mathrm{O}}_{2}^{-\bullet }$O2-∙ in Jin A (left) and Yamian A (right). (b) Content of H2O2 in Jin A (left) and Yamian A (right). (c) Content of MDA in Jin A (left) and Yamian A (right). Values are means ± SD of three replicates. Asterisks represent statistically significant differences between sterile line and its maintainer (* P < 0.05; ** P < 0.01, Student’s t tests). [file 12870_2023_4317_MOESM2_ESM.tif]
