# Supplementary material for: ROS accumulation-induced tapetal PCD timing changes leads to microspore abortion in cotton CMS lines
Source: BMC Plant Biol. 2023 Jun 12;23:311. doi: 10.1186/s12870-023-04317-5 (PMC10259065; doi:10.1186/s12870-023-04317-5)
Supplement: Supplementary file 3 — Additional file 3: Table 1. The relative expression of antioxidase genes by qRT-PCR. Values are means ± SD of three replicates. Asterisks represent statistically significant differences between sterile line and its maintainer (*P < 0.05; ** P < 0.01, Student’s t tests). GPX, Glutathione peroxidase; APX, Ascorbate peroxidase. [file 12870_2023_4317_MOESM3_ESM.docx]

|  |  | Jin B | Jin A | Yamian B | Yamian A |
| --- | --- | --- | --- | --- | --- |
|  | Stage 2 | 1.057±0.075 | 0.934±0.043 | 0.945±0.063 | 1.156±0.315 |
| *MnSOD* | Stage 3 | 1.040±0.166 | 1.161±0.054 | 1.060±0.095 | 1.176±0.253 |
|  | Stage 4 | 1.032±0.087 | 1.232±0.040* | 1.190±0.202 | 1.092±0.031 |
|  | Stage 2 | 2.429±0.187 | 2.888±0.220 | 2.392±0.294 | 4.126±0.575* |
| *Cu-ZnSOD* | Stage 3 | 4.011±0.316 | 3.537±0.402 | 4.124±0.432 | 6.118±0.153** |
|  | Stage 4 | 5.380±0.448 | 5.851±0.486 | 5.532±0.483 | 7.224±0.295* |
|  | Stage 2 | 0.266±0.015 | 0.168±0.010* | 0.346±0.075 | 0.311±0.040 |
| *FeSOD* | Stage 3 | 0.214±0.007 | 0.206±0.030 | 0.343±0.044 | 0.279±0.031 |
|  | Stage 4 | 0.296±0.064 | 0.180±0.034 | 0.311±0.034 | 0.302±0.029 |
|  | Stage 2 | 0.242±0.029 | 0.145±0.014* | 0.289±0.056 | 0.238±0.026 |
| *GPX6* | Stage 3 | 0.287±0.061 | 0.140±0.007* | 0.218±0.015 | 0.221±0.038 |
|  | Stage 4 | 0.313±0.040 | 0.177±0.020* | 0.286±0.060 | 0.232±0.026 |
|  | Stage 2 | 1.850±0.257 | 3.144±0.155** | 1.619±0.021 | 2.312±0.436 |
| *APX* | Stage 3 | 1.762±0.096 | 2.957±0.117** | 1.500±0.062 | 4.816±0.715** |
|  | Stage 4 | 2.315±0.165 | 3.153±0.051** | 2.104±0.093 | 3.859±0.097** |
|  | Stage 2 | 0.707±0.103 | 0.831±0.109 | 0.749±0.086 | 1.000±0.017** |
| *CAT1* | Stage 3 | 0.787±0.065 | 0.771±0.082 | 0.682±0.089 | 0.547±0.025 |
|  | Stage 4 | 1.325±0.082 | 1.175±0.063 | 0.763±0.174 | 0.366±0.032* |
|  | Stage 2 | 0.066±0.010 | 0.036±0.006* | 0.050±0.007 | 0.055±0.007 |
| *CAT2* | Stage 3 | 0.064±0.002 | 0.038±0.004** | 0.048±0.006 | 0.051±0.007 |
|  | Stage 4 | 0.053±0.006 | 0.054±0.008 | 0.045±0.003 | 0.054±0.009 |
