# Supplementary material for: ROS accumulation-induced tapetal PCD timing changes leads to microspore abortion in cotton CMS lines
Source: BMC Plant Biol. 2023 Jun 12;23:311. doi: 10.1186/s12870-023-04317-5 (PMC10259065; doi:10.1186/s12870-023-04317-5)
Supplement: Supplementary file 4 — Additional file 4: Table 2. The relative expression of RBOHE by qRT-PCR. Values are means ± SD of three replicates. Asterisks represent statistically significant differences between sterile line and its maintainer(* P < 0.05; ** P < 0.01, Student’s t tests). [file 12870_2023_4317_MOESM4_ESM.docx]

|  | Jin B | Jin A | Yamian B | Yamian A |
| --- | --- | --- | --- | --- |
| Stage 2 | 0 | 0.005±0.003 | 0.006±0.001 | 0 |
| Stage 3 | 0.014±0.001 | 0.006±0.002** | 0.017±0.002 | 0.004±0.001** |
| Stage 4 | 0.028±0.003 | 0.007±0.003** | 0.027±0.002 | 0.009±0.002** |
