# Supplementary material for: ROS accumulation-induced tapetal PCD timing changes leads to microspore abortion in cotton CMS lines
Source: BMC Plant Biol. 2023 Jun 12;23:311. doi: 10.1186/s12870-023-04317-5 (PMC10259065; doi:10.1186/s12870-023-04317-5)
Supplement: Supplementary file 5 — Additional file 5: Table 3. Primer sequence. [file 12870_2023_4317_MOESM5_ESM.docx]

| Name | [Sequence](javascript:;) | Size |
| --- | --- | --- |
| ef1α-F | agaccaccaagtactactgcac | 22 |
| ef1α-R | ccaccaatcttgtacacatcc | 21 |
| RBOHE-F | CGGAGGGAGTAGTTCCAAGC | 20 |
| RBOHE-R | CCAACCTGTTGTGGAGTCGT | 20 |
| CAT1-F | AGCTTGCATTTTGCCCTGC | 19 |
| CAT1-R | ATGAGCACACTTGGGAGCATT | 21 |
| CAT2-F | TCTTTTCGCACCATCCCGAA | 20 |
| CAT2-R | GTGGCATGACTGTGATTCGC | 20 |
| GPX6-F | TGTGAATGGCGAGAACGCAG | 20 |
| GPX6-R | AAAACATCAGCCAAGCAGTTTCT | 23 |
| APX-F | TGGCACTCAGCTGGAACTTT | 20 |
| APX-R | AAGCTGATAGAAGTCAGCGTATGA | 24 |
| MnSOD-F | GTTCAACGGCGGAGGTCA | 18 |
| MnSOD-R | TCAATGATTCCAATGAACCAAAGT | 24 |
| CuZnSOD-F | CGCCATGCTGGTGATCTAGG | 20 |
| CuZnSOD-R | ACCGCAAGCTACTCTACCAC | 20 |
| FeSOD-F | GAAAATGCAGTAAATCCTTG | 20 |
| FeSOD-R | TTAACTGTTTCCCAAGATAC | 20 |
